# Lie polynomials and a twistorial correspondence for amplitudes

**DOI:** 10.1007/s11005-021-01483-1

**Published:** 2021-12-05

**Authors:** Hadleigh Frost, Lionel Mason

**Affiliations:** The Mathematical Institute, University of Oxford, AWB, ROQ, Oxford, OX2 6GG UK

**Keywords:** Scattering amplitudes, Lie polynomials, Twistor theory, 81T13

## Abstract

We review Lie polynomials as a mathematical framework that underpins the structure of the so-called double copy relationship between gauge and gravity theories (and a network of other theories besides). We explain how Lie polynomials naturally arise in the geometry and cohomology of $\mathcal {M}_{0,n}$, the moduli space of *n* points on the Riemann sphere up to Mobiüs transformation. We introduce a twistorial correspondence between the cotangent bundle $T^*_D\mathcal {M}_{0,n}$, the bundle of forms with logarithmic singularities on the divisor *D* as the twistor space, and $\mathcal {K}_n$ the space of momentum invariants of *n* massless particles subject to momentum conservation as the analogue of space–time. This gives a natural framework for Cachazo He and Yuan (CHY) and ambitwistor-string formulae for scattering amplitudes of gauge and gravity theories as being the corresponding Penrose transform. In particular, we show that it gives a natural correspondence between CHY half-integrands and scattering forms, certain $n-3$-forms on $\mathcal {K}_n$, introduced by Arkani-Hamed, Bai, He and Yan (ABHY). We also give a generalization and more invariant description of the associahedral $n-3$-planes in $\mathcal {K}_n$ introduced by ABHY.

## Introduction

Colour-kinematics duality and the double copy [1, 2] have had a powerful influence on recent developments in scattering amplitudes. They stem from the KLT relations in string theory [3] between gravity and Yang–Mills tree-amplitudes and have been developed as a tool for the study of multiloop gravity amplitudes and more recently for applications to perturbative classical gravity calculations in connection with gravitational waves [4]. Notwithstanding the physical applications, the underlying mathematical framework is perhaps rather surprising even at tree level. There is little hint of such a double copy structure in standard approaches to perturbation theory of the classical nonlinear theories involved. The purpose of this article is to develop some of the underpinning mathematical structures. We build on observations by Kapranov in an after dinner talk [5] concerning the relevance of Lie polynomials, both in the double copy and in the Parke–Taylor expressions that pervade the subject. We also build on the recent work by Arkani-Hamed, Bai, He and Yan [6] that introduces differential forms in the space of kinematic invariants, $\mathcal {K}_n$. We tie them together by means of a double fibration correspondence that leads to a Penrose-like transform for the formulae of Cachazo He and Yuan (CHY) arising from the scattering equations [7, 8].

The first section provides an elementary review of the theory of Lie polynomials as relevant to this topic and expresses standard facts about the double copy in this language. In particular, the trivalent diagrams of BCJ are a representation of elements $\Gamma \in Lie(n-1)$, the space of Lie polynomials of degree $n-1$, and BCJ numerators $N_\Gamma$ are homomorphisms $N:Lie(n-1)\rightarrow V$ where *V* is some vector space of polynomials in the polarization data and momenta.

We next review the role played by Lie polynomials in the geometry of the moduli space $\mathcal {M}_{0,n}$ of *n* points $\sigma _i$ in $\mathbb {CP}^1$, both in describing the compact cycles in the homology $H_{n-3}(\mathcal {M}_{0,n}-D)$, which is isomorphic to $Lie(n-1)$, and dually the relative cocycles in $H^{n-3}(\mathcal {M}_{0,n},D)$, represented by the top degree holomorphic *Parke–Taylor forms*.

With these preliminaries in hand, we study a double fibration between the space of Mandelstam variables, $\mathcal {K}_n$, and $T^*_D\mathcal {M}_{0,n}$

1.1 $$\begin{aligned} ~&\mathcal {Y}_n=\mathcal {K}_n\times \mathcal {M}_{0,n},&(s_{ij},\sigma _j)\nonumber \\&p\swarrow \qquad \searrow q&\nonumber \\ (s_{ij}),~ \mathcal {K}_n&\mathbb {T}= T^*_D \mathcal {M}_{0,n}, ~ (\tau _i,\sigma _i) . \end{aligned}$$

where *p* forgets the second factor and and *q* is defined by the incidence relations

1.2 $$\begin{aligned} \tau _i =E_i(s_{kl}, \sigma _m):=\sum _j \frac{s_{ij}}{\sigma _{ij}}, \end{aligned}$$

which give the left hand side of the scattering equations. In the language of this correspondence, the CHY formulae are a Penrose transform, being simply the push down of the pullback of certain forms on $\mathbb {T}$. We investigate other more geometrical aspects of the Penrose transform. In particular, we show that the top power of the symplectic form $\omega ^{n-3}$ provides a correspondence between certain $(n-3)$-forms $w_\Gamma$ on $\mathcal {K}_n$ that were introduced by ABHY and homology classes in $\mathcal {M}_{0,n}$. ABHY use the $w_\Gamma$ as numerators so that given a set of conventional numerators *N* one can associate a *scattering *$(n-3)$*-form* $\Omega _N$. These arise from our double fibration via a Penrose transform also. Dually, ABHY introduce associahedral $(n-3)$-planes in $\mathcal {K}_n$ that can be used to convert a scattering form into a conventional amplitude. We give an improved and extended definition of these and show how they tie into the geometry of the correspondence.

## The double copy and Lie polynomials

Colour structures for *n*-point amplitudes are degree *n* invariant polynomials of weight one in each of the *n* Lie algebra ‘colours’ of the external particles. These naturally arise in Feynman rules as trivalent Feynman diagrams whose vertices are the structure constants of some unspecified Lie algebra. If we fix the *n*th particle, and an invariant inner product on the Lie algebra, at tree-level, such a polynomial can be realized as the inner product of the *n*th colour with the Lie algebra element with a *Lie polynomial* formed by successive commutators of the $n-1$ other colours working through the diagram back from the *n*th particle. This section reviews material concerning such colour structures in the language of free Lie algebras and Lie polynomials together with their duality with words formed from permutations of the $n-1$ labels of the first $n-1$ external particles. A classic text on free Lie algebras is [9].

### A review of words, Lie polynomials and trees

The space of words, $W({n-1})$, is the $(n-1)!$-dimensional linear span of words

$$a=x_{a(1)}x_{a(2)}\ldots x_{a(n-1)},$$

where the letters $x_{a(i)}\in \{x_1,\ldots ,x_{n-1}\}$ are all distinct, so that the *a*’s define permutations on $n-1$ letters. There is a natural bilinear inner product on $W(n-1)$ that is defined on monomials *a* and *b* by

$$\begin{aligned}(a,b):=\prod _{i=1}^{n-1}\delta _{a(i)b(i)},\end{aligned}$$

i.e. (*a*, *b*) is 1 if *a* and *b* are the same word, and 0 otherwise. A ‘Lie polynomial’ in $W(n-1)$ is any expression formed by taking $n-2$ iterated commutators of the $x_i$. An example is

$$\begin{aligned} \Gamma =[x_1,[\ldots ,[x_{n-1},x_n]\ldots ]] + [x_n,[\ldots ,[x_{n-2},x_{n-1}]\ldots ]], \end{aligned}$$

where $[x_i,x_j]$ is the commutator, $x_ix_j-x_jx_i$. Let $Lie(n-1)$ be the linear subspace of $W(n-1)$ generated by all Lie polynomials $\Gamma$ of weight $n-1$ in the $n-1$ variables, $x_1, \ldots , x_{n-1}$, with weight one in each. Every Lie monomial $\Gamma$ defines a rooted trivalent tree decorated by an orientation. We denote this tree also by $\Gamma$. An orientation of the tree can be presented as a planar embedding, where two planar embeddings have the same orientation if they differ from one another by an even number of flips. Thus, for example, the monomial $\Gamma = [2,[[1,3],[5,4]]]$ is associated with the following two planar embeddings, among others.


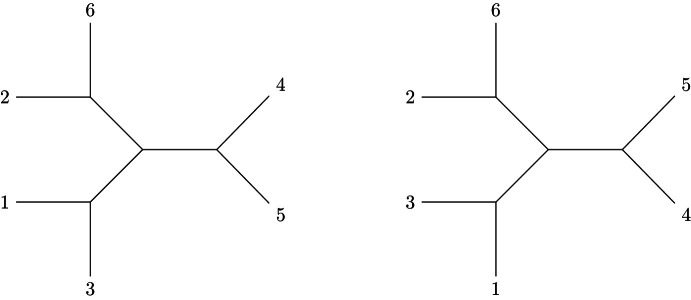


The Jacobi identity implies the vanishing of the sum of the three four-point oriented trees corresponding to an *s*, *t* and *u*-channel exchange graph. 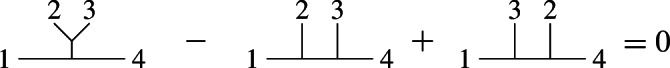


We will denote three Lie polynomials or corresponding graphs that differ only on such a four point subgraph by $\Gamma _s, \Gamma _t$ and $\Gamma _u$, and we will consequently have

2.1 $$\begin{aligned} \Gamma _s+\Gamma _t+\Gamma _u=0. \end{aligned}$$

The Lie monomial notation is useful for keeping track of the orientations of trees. Recall the inner product ( ,  ) defined on words. For a Lie monomial $\Gamma$, $(\Gamma ,a)$ is the coefficient of *a* in the expansion of its Lie monomial $\Gamma$. When $\Gamma$ is not planar for the ordering *a*, $(\Gamma ,a) = 0$. When $\Gamma$ is planar for the ordering *a*, $(\Gamma , a)$ is the orientation of that planar embedding, either $+1$ or $-1$. As an application of the notation, we can write

2.2 $$\begin{aligned} \Gamma =\sum _a (\Gamma ,a)a , \end{aligned}$$

which is simply the expansion of the commutators in $\Gamma$.

There are many characterizations of $Lie(n-1)$ as a subspace of $W(n-1)$ [9], and some of these have long been known in the physics literature as relations among gauge theory tree amplitudes. For instance, the U(1) decoupling identity is a consequence of Ree’s theorem.

#### Proposition 2.1

(Ree [10]) A polynomial $w\in W[n-1]$ is a Lie polynomial iff  for all nontrivial shuffles .^1^

This proposition implies that $Lie(n-1)^*$, the dual vector space of $Lie(n-1)$, can be understood as the quotient vector space $W(n-1)/\text {Sh}$, where $\text {Sh}$ is the subspace generated by all nontrivial shuffles.

#### Lemma 2.2

(Radford [11]) The $(n-2)!$ words of the form 1*a* are a basis for $Lie(n-1)^*$.

The direct expansion of $\Gamma _{1a}$ into words is2.3where |*u*| is the length of *u*. It follows that

$$\begin{aligned} (\Gamma _{1a},1b) = (a,b). \end{aligned}$$

In other words, we have the following

#### Lemma 2.3

The Kleiss–Kuijf (KK) basis of $Lie(n-1)^*$ given by words 1*a* is dual to the DDM basis of $Lie(n-1)$ given by combs $\Gamma _{1a}$.

An immediate consequence of this Lemma is that any $b+\text {Sh} \in W(n-1)/\text {Sh}$ may be expanded in this basis as

2.4 $$\begin{aligned} b+\text {Sh} = \sum _a (\Gamma _{1a},b) 1a + \text {Sh}. \end{aligned}$$

Dually, given that the combs are a basis for $Lie(n-1)$, a polynomial *w* is a Lie polynomial iff there is an expansion of *w* in the combs $\Gamma _{1a}$. Using Lemma 2.3, we find that

2.5 $$\begin{aligned} w = \sum (w,1a) \Gamma _{1a}. \end{aligned}$$

By Eq. (2.3), we find thatwhere $\bar{u}$ is the reversal of *u*. This identity implies the Kleiss–Kuijf relations, which we can restate as a theorem about Lie polynomials.

#### Proposition 2.4

(Kleiss–Kuijf) A polynomial $w\in W[n-1]$ is a Lie polynomial iff the Kleiss–Kuijf relations [12] hold:2.6

### The geometry of $\mathcal {K}_n$

Write $\mathcal {K}_n\simeq \mathbb {R}^{n(n-3)/2}$ for the vector space of Mandelstam variables. In coordinates $s_{ij}$, $i,j=1,\ldots , n$ (with $s_{ij}=s_{ji}$, $s_{ii}=0$), $\mathcal {K}_n$ is the hyperplane given by the equations

2.7 $$\begin{aligned} \sum _{j=1}^n s_{ij} = 0. \end{aligned}$$

In terms of null momenta, $k_i^\mu$, these variables are defined by $s_{ij} = 2 k_i\cdot k_j$, and so are subject to Gram determinant conditions. We ignore these Gram conditions and treat the variables $s_{ij}$ as independent.^2^ For amplitudes, the key geometric structure in $\mathcal {K}_n$ is the factorization hyperplanes given by $s_I=0$, where $I\subset \{1,2,\ldots ,n\}$ and

2.8 $$\begin{aligned} s_I:=\sum _{i,j\in I} s_{ij}=\left( \sum _i k_i\right) ^2. \end{aligned}$$

Let $\bar{I}$ be the complement of *I*, so that $s_{\bar{I}}=s_I$ by (2.7). Locality states that the only singularities of tree amplitudes are simple poles on these hyperplanes. A further requirement is that a double pole on the intersection of $s_I=0$ and $s_J=0$ occurs in an amplitude only if $I\subset J$ or $\bar{J}$. It follows that the allowed pole structure of a contribution to an *n*-point amplitude has poles along at most $n-3$ factorization hyperplanes $s_{I_p}=0$ for $p=1,\ldots ,n-3$. Such choices are in one-to-one correspondence with trivalent (and unoriented) trees.

### The double copy from biadjoint scalars to gauge and gravity theories

The double copy principle is that massless *n*-point tree amplitudes for a large web of important theories, including many gauge and gravity theories, can be expressed as a double copy in the form

2.9 $$\begin{aligned} \mathcal {M}=\sum _\Gamma \frac{N_\Gamma \tilde{N}_\Gamma }{d_\Gamma }. \end{aligned}$$

Here, the denominators

2.10 $$\begin{aligned} d_\Gamma =\prod _{r=1}^{n-3} s_{I_r} \end{aligned}$$

are the propagator factors associated with the graph $\Gamma$ thought of as a Feynman graph. Further, each trivalent diagram $\Gamma$ has a pair of *numerator* factors $N_\Gamma$ and $\tilde{N}_\Gamma$ that are functions of momenta, polarization data, flavour and colour. Such factors are said to be *local* if they are polynomial, i.e. admit no spurious singularities.

The key additional feature required to be a BCJ numerator is that $N_\Gamma$ and $\tilde{N}_\Gamma$ should represent homomorphisms from Lie polynomials to some vector space *V* of functions,

2.11 $$\begin{aligned} N:Lie(n-1)\rightarrow V. \end{aligned}$$

Thus, for any three graphs $\Gamma _s$, $\Gamma _t$ and $\Gamma _u$ satisfying $\Gamma _s+\Gamma _t+\Gamma _u=0$ as Lie polynomials, we must also have that

2.12 $$\begin{aligned} N_{\Gamma _s}+N_{\Gamma _t}+N_{\Gamma _u}=0. \end{aligned}$$

For this reason, the numerators are not uniquely determined: given a triple $\Gamma _s$, $\Gamma _t$ and $\Gamma _u$ we can perform the shift $\delta (N_{\Gamma _s}, N_{\Gamma _t}, N_{\Gamma _u})=(s,t,u)A$ for any $A\in V$. It also follows that BCJ numerators can be determined from their values on a comb basis $N_{\Gamma _{1a}}$ by

2.13 $$\begin{aligned} N_\Gamma =\sum _{a} (\Gamma ,1a) N_{\Gamma _{1a}}. \end{aligned}$$

In the case of Yang mills, the claim of BCJ [1] is that

2.14 $$\begin{aligned} \mathcal {A}=\sum _\Gamma \frac{N_\Gamma ^{k,\epsilon } c_\Gamma }{d_\Gamma } \end{aligned}$$

for some *kinematic numerators* $N_\Gamma ^{k,\epsilon }$ depending linearly on each polarization vector $\epsilon _i$ and rationally (or even polynomially) on the momenta satisfying (2.12). The key nontrivial output of the double copy is that gravity amplitudes are obtained when $\tilde{N}_\Gamma =N_\Gamma ^{k,\epsilon }$. The same numerators determine both the colour-ordered Yang–Mills amplitude with order *a* is then

2.15 $$\begin{aligned} \mathcal {A}_a = \sum _\Gamma \frac{N_\Gamma (\Gamma ,a)}{d_\Gamma } \end{aligned}$$

and gravity amplitudes by

2.16 $$\begin{aligned} \mathcal {M}= \sum _\Gamma \frac{N_\Gamma N_\Gamma }{d_\Gamma }. \end{aligned}$$

The most basic theory in this framework is the bi-adjoint scalar theory whose colour ordered amplitudes are given by

2.17 $$\begin{aligned} m(a,b):=\sum _\Gamma \frac{(\Gamma ,a)(\Gamma ,b)}{d_\Gamma } \end{aligned}$$

and we can introduce two underlying abstract amplitudes for these theories given by

2.18 $$\begin{aligned} m=\sum _\Gamma \frac{\Gamma \otimes \Gamma }{d_\Gamma }\in Lie(n-1)\otimes Lie(n-1) , \qquad m(a)=\sum _\Gamma \frac{(\Gamma ,a)\Gamma }{d_\Gamma }\in Lie(n-1) . \end{aligned}$$

Substituting (2.13) into (2.15), we obtain Yang–Mills amplitudes in terms of numerators and *m*(*a*, *b*) by

2.19 $$\begin{aligned} \mathcal {A}_a=(N,m(a))=\sum _b m(a,1b) N_{\Gamma _{1b}}^{k,\epsilon }\, , \end{aligned}$$

with a similar form for gravity

2.20 $$\begin{aligned} \mathcal {M}=(N\otimes \tilde{N}, m) =\sum _{a,b} m(1a,1b)N_{1a}^{k,\epsilon }\tilde{N}_{1b}^{k,\tilde{\epsilon }}. \end{aligned}$$

We briefly remark that the basic kinematic numerators $N^{k,\epsilon }_\Gamma$ for Yang Mills were obtained in [13]. Related numerators for other theories can be deduced from the Yang–Mills ones. The numerators $N^{k,\epsilon ,m}_\Gamma$ (see Table 1) can be related to $N^{k,\epsilon }_\Gamma$ by taking some components of the polarization vectors to be in a higher dimension to the momenta, as described (at the level of the CHY integrands) in [14]. The amplitudes for the theories in Table 1 are then given by

$$\begin{aligned} \text{ Amplitudes: } \qquad \mathcal {M}=\sum _\Gamma \frac{N_\Gamma ^l N_\Gamma ^r}{d_\Gamma }. \end{aligned}$$

See [4] for an up-to-date list of available numerators and their details.

**Table 1:** Theories arising from the different choices of numerators; see [14]

$N^l$	$N^r$			
	$N_\Gamma ^{k,\epsilon }$	$N_{\Gamma }^{k,k}$	$N_{\Gamma }^{k,\epsilon , m}$	$c_{\Gamma } \text{ or } (\Gamma ,a)$
$N_\Gamma ^{k,\epsilon }$	E			
$N_{\Gamma }^{k,k}$	BI	Galileon		
$N_{\Gamma }^{k,\epsilon ,m}$	${\mathop {\text {U}(1)^{m}}\limits ^{EM}}$	DBI	${\mathop {\text {U}(1)^{m}\times \text {U}(1)^{\tilde{m}}}\limits ^{EMS}}$	
$c_\Gamma \text{ or } (\Gamma ,a)$	YM	Nonlinear $\sigma$	${\mathop {\text {SU}(N)\times \text {U}(1)^{\tilde{m}}}\limits ^{EYMS}}$	${\mathop {\text {SU}(N)\times \text {SU}(\tilde{N})}\limits ^{\text {Biadjoint Scalar}}}$

We cannot simply invert (2.19) to obtain the $N_{1b}$ as *m*(1*a*, 1*b*) is not invertible. Indeed, all theories that can be expressed in this double-copy format with one explicit Lie polynomial factor satisfy the fundamental BCJ relations [1]. There are many forms of the relations, one version being, for a word^3^$a\in W(n-2)$2.21Thus, (2.19) determines the $N_{1a}$ only up to the addition of multiples of the BCJ relations. This freedom can be used to set all but $(n-3)!$ of the $N_{1a}$ to zero, but this is at the expense of requiring numerators that are rational rather than polynomial in the momenta, so that the remaining numerators will then have spurious poles.

### A note on the kinematic algebra

Given a Lie algebra, *g*, with an inner product, a Lie monomial $\Gamma \in Lie(n-1)$ gives rise to a ‘colour factor’, $c_\Gamma$, for every n-tuple of Lie algebra elements, $T_1,\ldots ,T_n$,

$$\begin{aligned} c_\Gamma : = \mathrm {tr}( \Gamma [T_1,\ldots ,T_{n-1}] T_n ). \end{aligned}$$

This means we have a map

$$\begin{aligned} c: Lie(n-1) \rightarrow \otimes ^n g^*, \end{aligned}$$

and it is a homomorphism, since the $c_{\Gamma }$ clearly satisfy the Jacobi identity. The BCJ kinematic numerators for Yang–Mills, $N^{k,\epsilon }_\Gamma$, likewise satisfy the Jacobi identity leading to a suggestion that they might arise from some *kinematic algebra*, an as yet unidentified Lie algebra. If that were the case, the double copy would be replacing the Yang–Mills Lie algebra numerator $c_\Gamma$ with kinematic numerators $N^{k,\epsilon }_\Gamma$. However, (2.12) does not imply that there is a Lie algebra, *g*, such that $N_\Gamma$ is the colour factor for that Lie algebra. This is clear for example in the case of $(\Gamma ,a)$ (and the forms $w_\Gamma$ below). Nevertheless, there has been some interesting work to identify such a Lie algebra associated with the kinematic numerators [16–18], (2.12).

In general, a homomorphism from $Lie(n-1)$ to a vector space of functions can be given by choosing any $(n-2)!$ such functions, as in Eq. (2.13). These have been identified in the case of Yang–Mills by various authors by recursion and in particular for Yang Mills in [19].

## Trees and words in $\mathcal {M}_{0,n}$

In this section, we consider the homology and cohomology of $\mathcal {M}_{0,n}$, the Deligne–Mumford compactification of the space of *n* distinct points on the Riemann sphere $\mathbb {CP}^1$ up to Möbius transformations. We first recall the basic properties of $\mathcal {M}_{0,n}$. $\mathcal {M}_{0,n}$ has a normal crossing divisor *D*, whose top dimensional strata are the codimension one components $D_I$, for $I\subset \{1,\ldots ,n\}$, where the points in *I* bubble off onto a new $\mathbb {CP}^1$, attached to the first by a node. 
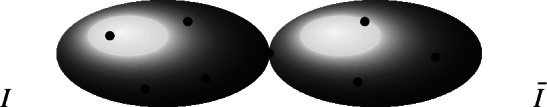
 Two such components, $D_I$ and $D_J$, intersect iff $I\subset J$ or $I\subset \bar{J}$. Then, $D_I\cap D_J$ corresponds to nodal curves with 3 components; for example when $I\subset J$, one containing the points *I*, the second $J-I$ and the third $\bar{J}$. It follows that the maximal intersections of these $D_I$ are points, $D_\Gamma \in \mathcal {M}_{0,n}$, given by the intersection of $n-3$ compatible $D_{I_p}$. Each such point corresponds to a nodal curve with $n-3$ components, each with three points that are either nodes or marked points. Such a tuple of compatible sets $I_p$ defines a trivalent tree, $\Gamma$, with the components corresponding to the vertices and nodes to propagators. The 0-dimensional strata of *D* are thus in one-to-one correspondence with trivalent (and unoriented) trees.

The complement of the divisor in $\mathcal {M}_{0,n}$ is an open top cell, $\mathcal {M}_{0,n}^\#:= \mathcal {M}_{0,n} - D$, on which we can use simplicial coordinates $\sigma _i$, $i = 1, \ldots ,n$, with the gauge fixing $(\sigma _1,\sigma _{n-1},\sigma _n) = (0,1,\infty )$. However, in order to study $\mathcal {M}_{0,n}$ in the neighbourhood of the divisor, it is useful to introduce dihedral coordinates, which are a set of cross-ratios of the points. There is one such set of coordinates for every dihedral structure [20]. Given an ordering, *a*, the associated dihedral coordinates are the cross-ratios

3.1 $$\begin{aligned} u_{ij}:= (a_ia_{j-1}|a_{i-1}a_j) = \frac{\sigma _{a_i\,a_{j-1}}\sigma _{a_{i-1}\, a_j}}{\sigma _{a_ia_j}\sigma _{a_{i-1}\, a_{j-1}}}, \end{aligned}$$

for $1<i+1<j$ and $\sigma _{ij} = \sigma _i - \sigma _j$. Each such cross-ratio $u_{ij}$ is associated with the cord, $(a_i,a_{j+1})$, of the n-gon labelled by the ordering, *a*. We will also denote $u_{ij}$ by $u_I$, where $I \subset \{1,\ldots ,n\}$ is the subset $\{a_i,\ldots ,a_{j-1}\}$ or its complement.

If the ordering *a* is compatible with the set $I\subset \{1,\ldots ,n\}$, then the divisor component $D_I$ can be seen in these coordinates as the locus of $u_I = 0$ [20]. The points $D_\Gamma$ in the divisor are, in dihedral coordinates, given as follows. Choose any ordering *a* such that $\Gamma$ is planar for *a* (i.e. $(\Gamma ,a)\ne 0$), with propagators given by the subsets $I_p \subset \{1,\ldots ,n-1\}$. In the dihedral coordinates associated with *a*, there are $n-3$ cross-ratios $u_{I_p}$ corresponding to the propagators of $\Gamma$. The point $D_\Gamma$ is then given, in these coordinates, by $u_{I_p}= 0$ for all $p=1,\ldots ,n-3$.

The $u_{I_p}$ form a good set of coordinates near $\Gamma$. Relations between such coordinate systems near different such points are obtained from the non-crossing identity

3.2 $$\begin{aligned} u_{ij}=1-\prod _{(k,l)\in (i,j)^c} u_{kl}, \end{aligned}$$

where for $k<l$, $(k,l)\in (i,j)^c$ means that the diagonal (*k*, *l*) of the polygon with vertices $\{1,\ldots ,n\}$ crosses the diagonal (*i*, *j*).

### The cohomology of $\mathcal {M}_{0,n}$ and Parke–Taylor forms

The dimensions of the cohomology groups of $\mathcal {M}_{0,n}$ are given by the Poincaré polynomial^4^

3.3 $$\begin{aligned} P(t) :=\sum _i \dim (H^i(\mathcal {M}^\#_{0,n}))\,t^i=\prod _{k=2}^{n-2} (1+kt). \end{aligned}$$

The cohomology ring is generated by the $\, \mathrm {d}\log \sigma _{ij}$ in the standard gauge fixing, subject to the quadratic relations

3.4 $$\begin{aligned} \frac{d\sigma _{ij}}{\sigma _{ij}} \wedge \frac{d \sigma _{jk}}{\sigma _{jk}} + \frac{d\sigma _{jk}}{\sigma _{jk}} \wedge \frac{d\sigma _{ki}}{\sigma _{ki}} + \frac{d\sigma _{ki}}{\sigma _{ki}} \wedge \frac{d\sigma _{ij}}{\sigma _{ij}} =0. \end{aligned}$$

This gives the dimension of $\Gamma (\Omega _D^1)$ as $\sum _{k=2}^{n-2}=n(n-3)/2$ as claimed earlier. It also follows that the top cohomology $H^{n-3}(\mathcal {M}_{0,n},D)\simeq \Gamma (\mathcal {M}_{0,n},\Omega ^{n-3}_D)$ has dimension $(n-2)!$. A natural spanning set for $\Gamma (\Omega ^{n-3}_D)$ is provided by the Parke–Taylor forms

3.5 $$\begin{aligned} PT(123\ldots n)=\frac{1}{\mathrm {Vol}SL(2)} \bigwedge _{i=1}^n \frac{d\log \sigma _{i\, i+1}}{2\pi i}. \end{aligned}$$

In our gauge fixing

3.6 $$\begin{aligned} \frac{1}{\mathrm {Vol}SL(2)}=\frac{(2\pi i)^3\sigma _{1\, n-1}\sigma _{n-1\, n}\sigma _{n1}}{d\sigma _1d\sigma _{n-1} d\sigma _n} \end{aligned}$$

yielding now for a general choice of permutation *a* of $1,\ldots ,n-1$

3.7 $$\begin{aligned} PT_a:=PT(a n)=\frac{d^{n-3}\sigma }{\prod _{i=1}^{n-2} \sigma _{{a(i)\, a(i+1)}}} , \qquad d^{n-3}\sigma :=\frac{1}{(2\pi i)^{n-3}}\bigwedge _{i=2}^{n-2} d\sigma _i. \end{aligned}$$

The $(n-1)!$ Parke–Taylor forms defined in this way are not linearly independent, because they satisfy the shuffle relations of 2.4,3.8for *b*, *c* nontrivial, identically^5^ [23]. Thus, following Proposition 2.4, we deduce that

3.9 $$\begin{aligned} H^{n-3}(\mathcal {M}_{0,n},D)\simeq \Gamma (\mathcal {M}_{0,n},\Omega ^{n-3}_D) \simeq Lie(n-1)^*. \end{aligned}$$

Moreover, by Lemma 2.2, one can take a KK basis for $H^{n-3}(\mathcal {M}_{0,n},D)$, given by the $PT_{1a}$, for all $(n-2)!$ permutations *a*. It follows from Eq. (2.4) that we have the following identity.

#### Lemma 3.1

The forms $PT_a$ satisfy

$$\begin{aligned} PT_a = \sum _b (\Gamma _{1b},a) PT_{1b}. \end{aligned}$$

For future reference, observe that we can write $PT_{1a}$ in dihedral coordinates as

3.10 $$\begin{aligned} PT_{1a}=\mathrm {sgn}(a) \,\bigwedge _{i=3}^{n-1} \frac{1}{2\pi i}d\log \frac{ u_{1a_i}}{1- u_{1a_i}}, \end{aligned}$$

since, in the standard gauge fixing,

$$\begin{aligned} d\log \frac{ u_{1a_i}}{1- u_{1a_i}} = d\sigma _{a_{i-1}} \frac{\sigma _{1a_i}}{\sigma _{1a_{i-1}}\sigma _{a_{i-1}a_i}} + d\sigma _{a_i} \frac{1}{\sigma _{a_{i-1}a_i}}. \end{aligned}$$

In fact, for any tree $\Gamma$ compatible with the ordering *a* we have an associated top form,

3.11 $$\begin{aligned} PT_\Gamma := \bigwedge _{i=1}^{n-3} \frac{1}{2\pi i} d \log \frac{u_{I_p}}{1-u_{I_p}}, \end{aligned}$$

where the $I_p$ are (some ordering of) the subsets defining the propagators of $\Gamma$. In particular, $PT_{\Gamma _{1a}} = PT_{1a}$. $PT_\Gamma$ is not always equal to a standard Parke–Taylor for the given dihedral structure. For the ordering 1*a*, an example of a tree, $\Gamma$, such that $PT_{\Gamma } \ne PT_{1a}$ is the ‘snowflake’, $\Gamma = [[[1,2],[3,4]],5]$, for which one finds

$$\begin{aligned} PT_\Gamma = PT_{12345} \times (13|24). \end{aligned}$$

However, for a tree $\Gamma$ in which every vertex is attached to at least one external particle, $PT_\Gamma$ does give rise to the ordinary Parke–Taylor for that dihedral structure, as observed first in Koba and Nielsen in [24]. Such a $\Gamma$ (with every vertex connected to an external particle) corresponds to a quiver without cycles, as discussed in [25].

Finally, note that $\Gamma \mapsto PT_\Gamma$ is not a homomorphism from $Lie(n-1)$ to $H^{n-3}(\mathcal {M}_{0,n},D)$, since the $PT_\Gamma$ do not satisfy the Jacobi identity, which in particular implies that Eq. (2.5) cannot be used to expand $PT_\Gamma$ in a basis of $PT_a$’s.

### Homology of $\mathcal {M}_{0,n}$

We saw, in Eq. (3.9), that there is an isomorphism of $H^{n-3}(\mathcal {M}_{0,n},D)$ with $Lie(n-1)^*$. Integration gives a perfect pairing between relative cohomology, $H^{n-3}(\mathcal {M}_{0,n},D)$, and the homology of the complement of the divisor, $H_{n-3}(\mathcal {M}_{0,n} - D)$ [26]. It follows that the homology $H_{n-3}(\mathcal {M}_{0,n} - D)$ can be identified (as a vector space) with $Lie(n-1)$.^6^ In this section, we will review the description of the homology cycles of $\mathcal {M}_{0,n}-D$.

A point $D_\Gamma$ naturally gives rise to a class in $H_{n-3}(\mathcal {M}_{0,n}^\#)$ represented by a real half-dimensional torus that surrounds $D_\Gamma$. Explicitly, the cycle can be defined as the locus

3.12 $$\begin{aligned} C_\Gamma : |u_{I_p}|= \epsilon _p,\qquad p=1,\ldots ,n-3 \end{aligned}$$

for some small $\epsilon _p$ and choice of orientation. These cycles were first described in [27], and they generate the homology, but are not independent.^7^ Integrating a holomorphic top-form with $C_\Gamma$ evaluates the residue at $D_\Gamma$ [29]. Lemma 7.1 of [20] states that $PT_{a}$ can only have a pole at those $D_\Gamma$ which are compatible with the ordering *a*. Moreover, it is clear that we can orient the $C_{\Gamma _a}$ so that

$$\begin{aligned} \int _{C_{\Gamma _a}} PT_a = +1, \end{aligned}$$

which leads to the following,

#### Lemma 3.2

The cycles $C_{\Gamma _{1a}}$ represent a basis for $H_{n-3}(\mathcal {M}_{0,n}^\#)$, dual to the KK basis of $H^{n-3}(\mathcal {M}_{0,n},D)$:

$$\begin{aligned} \int _{C_{\Gamma _{1a}}}PT_{1b} = (1a,1b), \end{aligned}$$

where we have chosen to orient the $C_{\Gamma _{1a}}$ using the $PT_{1a}$ forms so that the residues are $+1$.

The relations among the top cycles are given by Jacobi-type relations.

#### Lemma 3.3

(Cohen^8^) For three trees related by Jacobi, there exists a contraction of the sum of the corresponding cycles, $C_{\Gamma _s}+C_{\Gamma _t}+C_{\Gamma _u}$.

An explicit homotopy contracting $C_{\Gamma _s}+C_{\Gamma _t}+C_{\Gamma _u}$ is easy to visualize for $n=4$, since $\mathcal {M}^\#_{0,4}=\mathbb {CP}^1-\{0,1,\infty \}$, with boundary points $D_{s}=0$, $D_{t}=1$, and $D_{u}=\infty$. Appropriately oriented, the three small circles around these points add up to zero in homology.


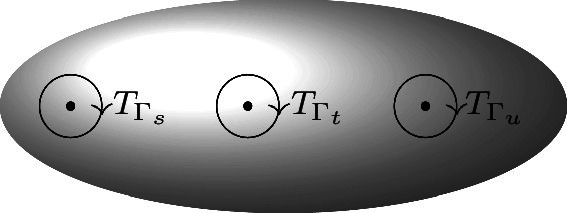


More generally, for $n>4$, we can exhibit a contraction of $C_{\Gamma _s}+C_{\Gamma _t}+C_{\Gamma _u}$ by making small all $\epsilon$’s in definition of the cycle, Eq. (3.12), except for the $\epsilon$ corresponding to the propagator being exchanged. This restricts us to a $\mathbb {CP}^1$ component of *D*, where the same argument made for $n=4$ can be applied.^9^

It follows from Lemma 3.3 and Eq. (2.5) that,

$$\begin{aligned} C_\Gamma = \sum _{a} (\Gamma ,1a)\Gamma _{1a}. \end{aligned}$$

Combining this with Lemmas 3.1 and 3.2 implies,

#### Lemma 3.4

The integration pairing between $C_\Gamma$ and $PT_{a}$ is

$$\begin{aligned}\int _{C_{\Gamma }} PT_{a} = (\Gamma ,a).\end{aligned}$$

## A Penrose transform for amplitudes

Our starting point is the observation that $\mathcal {K}_n$ can be identified with $H^1(\mathcal {M}_{0,n},D)$. Our ‘twistor space’ for the Penrose transform will be $\mathbb {T}=T^*_D \mathcal {M}_{0,n}$, the total space of the bundle of holomorphic 1-forms on $\mathcal {M}_{0,n}$ with logarithmic singularities on *D*. The relationship with $\mathcal {K}_n$ is given by the isomorphism

4.1 $$\begin{aligned} \mathcal {K}_n:=H^1(\mathcal {M}_{0,n},D) \simeq \Gamma ({\mathcal {M}_{0,n}},T^*_D ). \end{aligned}$$

This correspondence can be expressed by considering $d \log$ of the Koba–Nielsen factor [24], which is, in the standard gauge fixing,

4.2 $$\begin{aligned} KN:=\prod _{i<j} \sigma _{ij}^{s_{ij}}, \qquad \sigma _{ij}=\sigma _i-\sigma _j. \end{aligned}$$

This gives the general section of $\tau \in \Gamma (T^*_D \mathcal {M}_{0,n})$ as

4.3 $$\begin{aligned} \tau =\sum _iE_id\sigma _i:= \sum _{i<j} s_{ij}d \log \sigma _{ij}, \qquad E_i=\sum _j\frac{s_{ij}}{\sigma _{ij}}. \end{aligned}$$

The second equality shows that this is clearly invariant under translations and rescalings of the $\sigma _i$, but full Mobius invariance (i.e. vanishing when contracted with $\sum _i \sigma _i^2\partial _{\sigma _i}$) requires $\sum _i s_{ij}=0$. Our normalizations $(\sigma _1,\sigma _{n-1},\sigma _n)=(0,1,\infty )$ give the triviality of $d\log \sigma _{in}$ and $d\log \sigma _{1\,n-1}$ giving the correct dimensionality of the $d\log \sigma _{ij}$ basis of $H^1$. Note that the equations $E_i=0$ are the *scattering equations*.

To more clearly demonstrate the $d\log$ behaviour on *D*, given a choice of the standard ordering, we can also represent the Koba–Nielsen factor as [31]

4.4 $$\begin{aligned} KN=\prod _{j>i+1} u_{ij}^{X_{ij}}, \qquad X_{ij}=\sum _{i\le l<m<j} s_{lm}. \end{aligned}$$

This gives the useful representation of the general section in terms of the $n(n-3)/2$ basis $d\log u_{ij}$

4.5 $$\begin{aligned} \sum _iE_id\sigma _i=\sum _{j>i+1 }X_{ij} d \log u_{ij}. \end{aligned}$$

This representation manifests the $d\log$ behaviour on the components of *D* compatible with this choice of ordering.

### The double fibration and the CHY formulae

The twistor correspondence arises from the following double fibration:

4.6 $$\begin{aligned} ~&\mathcal {Y}_n=\mathcal {K}_n\times \mathcal {M}_{0,n},&(s_{ij},\sigma _j)\nonumber \\&p\swarrow \qquad \searrow q&\nonumber \\ (s_{ij}),~ \mathcal {K}_n&\mathbb {T}= T^*_D \mathcal {M}_{0,n}, ~ (\tau _i,\sigma _i) . \end{aligned}$$

where *p* forgets the second factor and *q* is defined by the incidence relations

4.7 $$\begin{aligned} \tau _i =E_i(s_{kl}, \sigma _m):=\sum _{j\ne i} \frac{s_{ij}}{\sigma _{ij}}. \end{aligned}$$

A point in $\mathcal {K}_n$ therefore determines a section $\tau _i=E_i$ of $\mathbb {T}\rightarrow \mathcal {M}_{0,n}$.

A special role is played by the zero-section $\mathbb {T}_0$ of $\mathbb {T}$ as it encodes the scattering equations; given generic $s_{ij}$, the section $\tau _i=E_i(\sigma )$ intersects $\mathbb {T}_0$ at the $(n-3)!$ solutions to the scattering equations. We therefore introduce $\bar{\delta }(\tau )^{n-3}$ to be the $(0,n-3)$-form delta function supported on $\mathbb {T}_0$. In the standard gauge fixing above, it can be defined by

4.8 $$\begin{aligned} \bar{\delta }(\tau )^{n-3}:=\prod _{i=2}^{n-2} \bar{\partial }\frac{1}{2\pi i\tau _i}, \qquad \text{ where } \qquad \bar{\partial }\frac{1}{2\pi i z}= \delta (\mathfrak {R}z) \delta (\mathfrak {I}z) d\bar{z}. \end{aligned}$$

More invariantly, this takes values in $(\Omega ^{n-3}_D\mathcal {M}_{0,n})^*$ so that to use it in an integrand, we will need an extra factor with values in $(\Omega ^{n-3}_D\mathcal {M}_{0,n})^2$.

A first observation is that the CHY formulae can be regarded as examples of a Penrose transform in the sense that the amplitudes are obtained as the pushdown to $\mathcal {K}_n$ of a pullback of an object from $\mathbb {T}$. The generic CHY formula takes the form:

4.9 $$\begin{aligned} \mathcal {M}(s_{ij},\ldots )=\int _{\mathcal {M}_{0,n}=p^{-1}(s_{ij})} q^*\left( \mathcal {I}_l \mathcal {I}_r \,\bar{\delta }(\tau )^{n-3}\right) \end{aligned}$$

Here, $\mathcal {I}_l, \mathcal {I}_r \in \Omega ^{n-3}_D\mathcal {M}_{0,n}$ are CHY half-integrands but also often depending also on polarization data, with the most basic example being *m*(*a*, *b*) when $(\mathcal {I}_l,\mathcal {I}_r)=(PT_a,PT_b)$. There is an empirical direct correspondence between choices of $I_{l/r}$ and numerators $N_\Gamma$ with for example the CHY Pfaffian^10^$\mathrm {Pf}'(M)$ corresponding to the $N^{k,\epsilon }_\Gamma$ described earlier. See [14, 32] for details of half-integrands for other theories and their origins.

### The geometry of the correspondence

A generic point $(\tau _i,\sigma _i)$ of $\mathbb {T}$ corresponds to a codimension-$n-3$ plane in $\mathcal {K}_n$. This plane is the $(n-2)(n-3)/2$ dimensional space of sections that pass through the point. For a point lying in a top-stratum, $D_I$, of the divisor, these planes lie inside the factorisation hyperplane plane $s_I=0$. This follows from the following combination [33] of the scattering equations

4.10 $$\begin{aligned} E_I=\frac{s_I}{2} + \sum _{i\in I, j\in \bar{I}} s_{ij} \frac{\sigma _{i1}\sigma _{jn}}{\sigma _{ij}\sigma _{1n}}. \end{aligned}$$

and the fact that, assuming $1\in I$ and $n\in \bar{I}$, the second term vanishes when restricted to $D_I$.

It follows that the point $(\tau _i,\sigma _i)=(0,D_\Gamma )\in \mathbb {T}$ corresponds to the codimension-$n-3$ plane in $\mathcal {K}_n$ given by the intersection of the planes $s_{I_p}=0$, where $I_p$ are the subsets of $\{1, \ldots ,n-1\}$ corresponding to the momentum flowing through each propagator in $\Gamma$. We can characterize these planes as being those planes passing through the origin with normal $n-3$-form

4.11 $$\begin{aligned} w_\Gamma = \pm \bigwedge _{p=1}^{n-3} d s_{I_p}. \end{aligned}$$

In the next section, we will see how the $w_\Gamma$ arise from the double fibration and show how their signs are fixed to make them satisfy the same relations as the $n-3$-forms defined by [6].

### The symplectic form and the holomorphic volume form

By studying the symplectic volume form $\omega ^{n-3}$ on $T^*_D\mathcal {M}_{0,n}$, we find two elementary consequences of the double fibration. In this section, we describe how the symplectic volume gives rise to a transform between $n-3$ cycles in $\mathcal {M}_{0,n}-D$ and the $n-3$-forms, Eq. (4.11), encountered above. In the next section, we will describe the associated correspondence between $(n-3)$-planes in $\mathcal {K}_n$ and $(n-3)$-cycles in $H^{n-3}(\mathcal {M}_{0,n},D)$. Distinguished classes in $H^{n-3}$ are seen to correspond to the planes defined by [6].

The symplectic form on $T^*_D\mathcal {M}_{0,n}$ can be written explicitly as

$$\begin{aligned} \omega = \sum _{i\ne 1,n-1,n} d\tau _i \wedge d\sigma _i, \end{aligned}$$

where $\tau _i$ are the components of $\tau = \sum \tau _id\sigma _i$ in these coordinates. Pulling back $\omega ^{n-3}$ to $\mathcal {Y}_n$, we can decompose it into a sum over a basis of $\Gamma (\mathcal {M}_{0,n},\Omega ^{n-3}_D)$ with coefficients given by $n-3$-forms on $\mathcal {K}_n$. This gives rise to a correspondence between $n-3$-forms on $\mathcal {K}_n$ and $(n-3)$-cycles in $\mathcal {M}_{0,n}$ which we explain in this section. In the next section, we describe the associated correspondence between $(n-3)$-planes in $\mathcal {K}_n$ (‘ABHY planes’) and $(n-3)$-cocycles in $H^{n-3}(\mathcal {M}_{0,n},D)$.

Every cycle $C_\Gamma$ in $H_{n-3}(\mathcal {M}_{0,n}-D)$ defines an $n-3$-form on $\mathcal {K}_n$,

4.12 $$\begin{aligned} w_\Gamma :=\int _{C_\Gamma } q^* \omega ^{n-3} \in \Omega ^{n-3}(\mathcal {K}_n)\, . \end{aligned}$$

It is clear that, for a Jacobi triple of trees,

4.13 $$\begin{aligned} w_{\Gamma _s}+w_{\Gamma _t}+w_{\Gamma _u}=0. \end{aligned}$$

In other words, the map $Lie(n-1)\hookrightarrow \wedge ^{n-3}\mathcal {K}_n^*$ given by $\Gamma \mapsto w_\Gamma$ is a homomorphism.

To find an explicit expression for $w_\Gamma$, we use the representation (4.5) of $q^*\tau$ in a choice of dihedral coordinates $u_{ij}$ for which $\Gamma$ is planar. This gives

4.14 $$\begin{aligned} q^*\omega =d(q^*\tau )=\sum _{i+1<j} d X_{ij}\wedge \frac{du_{ij}}{u_{ij}} \end{aligned}$$

It is then easily seen that integration of $q^*\omega ^{n-3}$ over $C_\Gamma$ picks out only those poles that correspond to propagators of $\Gamma$. The residue thus gives a wedge product $\wedge ds_{I_p}$, with the overall sign determined by the orientation of $C_\Gamma$. Given this explicit form of $w_\Gamma$, the Jacobi relation, Eq. (4.13), can also be understood to follow from the momentum conservation relation, $ds+dt+du=0$ [6].

It follows from Eq. (2.5) that $w_\Gamma$ admits the expansion

4.15 $$\begin{aligned} w_\Gamma =\sum _{a\in S_{N-2}} w_{\Gamma _{1a}} (\Gamma ,1a). \end{aligned}$$

Combining this with Lemmas 3.4 and 2.3, we find that we can write the pull-back of the symplectic volume form as

4.16 $$\begin{aligned} q^*\omega ^{n-3}=\sum _{a\in S_{n-2}} w_{\Gamma _{1a}} PT_{1a}. \end{aligned}$$

Although we have used the dual comb and KK bases, this relation follows in any dual basis, because $w_\Gamma$ and $PT_a$ furnish representations of $Lie(n-1)$ and $Lie(n-1)^*$, respectively, so that(4.16) is an explicit of writing the Kronecker delta. For example, when $n=4$, $q^*\omega$ can be written in any one of the three KK bases,

4.17 $$\begin{aligned} q^* \omega&= -ds_{12} \wedge PT_{213} - ds_{23} \wedge PT_{231} \nonumber \\&= ds_{12}\wedge PT_{123} + ds_{13}\wedge PT_{132}\nonumber \\&= -ds_{23}\wedge PT_{321} + ds_{13}\wedge PT_{312}. \end{aligned}$$

More generally, we can rewrite $q^*\omega ^{n-3}$ using whatever bases we choose.

#### Lemma 4.1

The pullback of the symplectic volume form to $\mathcal {Y}_n$ can be written

$$\begin{aligned} q^*\omega ^{n-3}=\sum _{\Gamma \in H, a\in K} (\Gamma ,a)\, w_{\Gamma } \wedge PT_{a}. \end{aligned}$$

for a basis *H* of $Lie(n-1)$, and a basis *K* of $Lie(n-1)^*$.

**Fig. 1:**
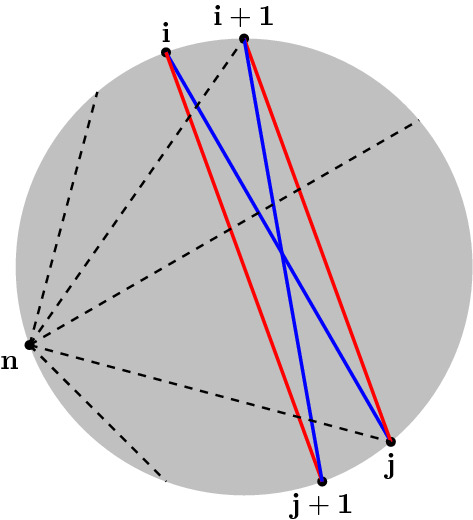
The function $s_{ij} = X_{ij}+X_{i+1j+1} - X_{ij+1} - X_{i+1j}$ is annihilated by the derivatives $D_I$ for each arc, *I*, from the point *n* to each one of $2,3,...,n-2$. This can be verified for each of the five cases indicated by the dashed lines in the figure. This is used in the main text to show that the $n-3$-plane spanned by the derivatives $D_I$ are also given by linear equations of the form $s_{ij} = \text {constant}$, for $1\le i< j-1 < n-2$

### Associahedral $(n-3)$-planes in $\mathcal {K}_n$ and forms on $\mathcal {M}_{0,n}$.

An alternative way to study the correspondence is to restrict $\omega ^{n-3}$ to different $(n-3)$-planes in $\mathcal {K}_n$. This correspondence is related to the construction in [34], although they discuss different planes and polytopes. Distinguished classes in $H^{n-3}(\mathcal {M}_{0,n},D)$ correspond to the associahedral planes found in [6].

An $n-3$-plane in $\mathcal {K}_n$ is defined up to translation by its tangent form $P \in \wedge ^{n-3}\mathcal {K}_n$. For any such plane, *P*, the symplectic volume form gives rise to an $n-3$-form in $\Gamma (\mathcal {M}_{0,n},\Omega _D^{n-3})$,Recall the definition of $PT_\Gamma \in \Gamma (\mathcal {M}_{0,n},\Omega _D^{n-3})$, given in Eq. (3.11), which is a top form associated with a dihedral structure, *a*, together with a tree $\Gamma$ that is compatible with that ordering. $PT_\Gamma$ corresponds to a plane $P_\Gamma$ in $\mathcal {K}_n$ defined as follows. Fix a dihedral structure *a* and let

$$\begin{aligned}D_{I}:=\frac{\partial }{\partial X_{I}}-\sum _{J\in I^c}\frac{\partial }{\partial X_{J}},\end{aligned}$$

such that we have

4.18 $$\begin{aligned} D_I \log KN= \log u_I -\sum _{J\in I^c} \log u_J= \log \frac{u_I}{1-u_I}, \end{aligned}$$

by the non-crossing identity, Eq. (3.2). It follows that, for a compatible set of propagators $I_p$, defining a tree $\Gamma$ that is compatible with *a*, we can define a plane

$$\begin{aligned} P_\Gamma = \bigwedge _{p=1}^{n-3}D_{I_p}, \end{aligned}$$

for some ordering of the propagators chosen so that4.19In particular, when we take $P_{a, \Gamma }$ to be the comb, $P_{1a}:=P_{1a,\Gamma _{1a}}$, we recover the Parke–Taylor factors in the standard KK basis,For a general $\Gamma$, in dihedral structure *a*, the equations of the plane associated with $P_\Gamma$ can be written as

4.20 $$\begin{aligned} X_J + \sum _{r \text { s.t. } J\in I_r^c} X_{I_r}=\text {const.}, \end{aligned}$$

for all *J* compatible with the ordering *a* and not corresponding to a propagator of $\Gamma$. These equations bear no obvious resemblance to those defined in [6], but we will see that our planes are the same as theirs.

First, notice that the $P_a$ satisfy

$$\begin{aligned} P_a = \sum (\Gamma _{1b},a)P_{1b}, \end{aligned}$$

by Lemma 3.1, and that, moreover,

$$\begin{aligned} (w_\Gamma ,P_a) = \int _{C_\Gamma } PT_a = (\Gamma , a), \end{aligned}$$

using Lemma 3.4.

In [6], the ABHY planes are defined, for the ordering $a=1\ldots n-1$ and the comb $\Gamma _{a}$, to be defined by the $(n-2)(n-3)/2$ equations

$$\begin{aligned} s_{ij}=\text {const.}, \end{aligned}$$

for $1\le i< j - 1 < n - 2$. Using the identity, $s_{ij} = X_{ij}+X_{i+1j+1}-X_{ij+1}-X_{i+1j}$ we can verify that, for $I_p$ in $\Gamma _a$,

4.21 $$\begin{aligned} D_{I_p} s_{ij} = 0, \end{aligned}$$

for all $1\le i< j - 1 < n - 2$, and each *p*. Fixing an $s_{ij}$, one can check this equation for each *p*. The chord $I_p$ is the arc *kn* for $k=2,\ldots ,n-2$. The five cases to check are (i) $k\le i$, (ii) $k=i+1$, (iii) $i+1<k<j$, (iv) $k=j$, and (v) $k>j$. That Eq. (4.21) vanishes in these five cases is easily seen from Fig. 1. Conversely, we can define the ABHY plane for the ordering *a* and the comb $\Gamma _a$ to be given by the tangent form

4.22 $$\begin{aligned} P_{a }=\bigwedge _{i=2}^{n-2}\left( \frac{\partial }{\partial s_{a_{i-1}\, a_i}}-\frac{\partial }{\partial s_{a_i\,a_{i+1}}}\right) . \end{aligned}$$

For each factor in $P_a$, we can check that it annihilates the expressions in Eq. (4.20). We have the following property

4.23 $$\begin{aligned} \left( \frac{\partial }{\partial s_{i-1\, i}}-\frac{\partial }{\partial s_{i\,i+1}}\right) X_{jk}=\left\{ \begin{array}{ll} 1, &{}\quad i=k-1,\\ -1, &{}\quad i=j,\\ 0, &{}\quad \text{ otherwise. } \end{array}\right. \end{aligned}$$

**Fig. 2:**
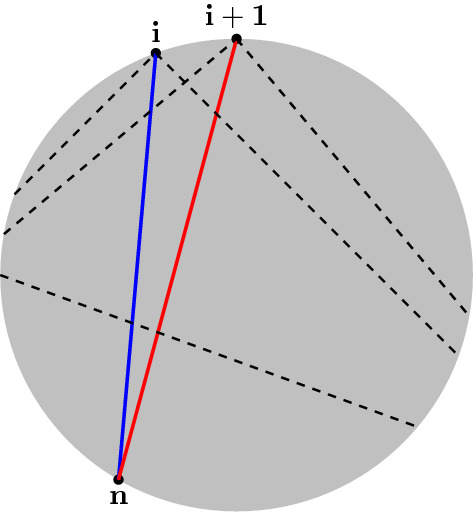
The functions given by (4.20) are annihilated by the derivatives $\partial / \partial s_{i-1i} - \partial / \partial s_{ii+1}$. This can be verified by considering the arc *J* in each of the five cases indicated by the dashed lines in the figure. In the text this leads to the result that the planes spanned by the vectors making up the $P_a$ in Eq. (4.22), are also given by Eq. (4.20)

For fixed *i*, there are five cases to check, and these are shown in Fig. 2. By these arguments, we conclude that

#### Lemma 4.2

The planes $P_{a,\Gamma }$ defined by the correspondence, Eq. (4.19), are the ABHY planes when $\Gamma _a$ is the comb for the ordering *a*.

Consider moreover any triangulation $\Gamma$ that has no internal triangles. I.e. every diagonal in the triangulation belongs to a ‘skinny’ triangle like the one illustrated in Fig. 2 (or, dually, every vertex in the tree $\Gamma$ is connected to an external line). In this case, the same arguments that lead to Lemma 4.2 can be applied, arc by arc, to show that:

#### Lemma 4.3

The planes $P_{a,\Gamma }$ defined by the correspondence, Eq. (4.19), are given by the $(n-2)(n-3)/2$ equations

$$\begin{aligned} s_{ij} = \text {const.}, \end{aligned}$$

for all chords (*ij*) compatible with the ordering that are not one of the $n-3$ propagators of $\Gamma$.

It is a consequence of the results in [35] that the planes in Lemma 4.3 cut out associahedra when intersected with the positive orthant $\mathcal {K}_n^+$. The planes are called ‘generalized ABHY’ planes: when $\Gamma$ is the comb, the planes are the same as the original ones in [6], and when $\Gamma$ is another tree (but with every vertex connected to an external particle) the plane $P_{a,\Gamma }$ is one of the ‘generalized ABHY’ planes whose equations are presented in [36].

### Scattering forms and CHY

The scattering forms of [6] are defined by the following sum over trees,

4.24 $$\begin{aligned} \Omega _a=\sum _{\Gamma }\frac{w_\Gamma (a,\Gamma )}{d_\Gamma } \, , \end{aligned}$$

where $d_\Gamma =\prod _{p=1}^{n-3} s_{I_p}$ and $I_p$ are the propagators of $\Gamma$. The $(a,\Gamma )$-factor reduces the sum to one over trees that are planar for the ordering *a*.

The symplectic form discussed above gives rise to the ABHY scattering forms on $\mathcal {K}_n$ from the Dolbeault formula

4.25 $$\begin{aligned} \Omega _a = \int _{p^{-1}(s_{ij})} q^* \left[ \bar{\delta }^{n-3}(\underline{\tau }) \wedge \omega ^{n-3} PT_a \right] , \end{aligned}$$

where

4.26 $$\begin{aligned} \bar{\delta }^{n-3}(\underline{\tau })=\bigwedge _{i=1}^{n-3} \bar{\delta }(\tau _i), \qquad \bar{\delta }(z)=\frac{1}{2\pi i} \bar{\partial }\frac{1}{z}. \end{aligned}$$

To see that Eq. (4.25) is equal to Eq. (4.24), we use Lemma 4.1 to rewrite Eq. (4.25) as

$$\begin{aligned} \Omega _a = \sum _b w_{\Gamma _{1b}} \int _{p^{-1}(s_{ij})} q^* \left[ \bar{\delta }^{n-3}(\underline{\tau }) \wedge PT_{1b} PT_a \right] . \end{aligned}$$

We recognize that the integral is the CHY formula [7, 8] for *m*(*a*, *b*), defined in Eq. (2.17). It follows that Eq. (4.25) can be expanded as

$$\begin{aligned} \Omega _a = \sum _b \frac{w_{\Gamma _{1b}} (\Gamma ,1b)(\Gamma ,a)}{d_\Gamma }, \end{aligned}$$

which is equal to (4.24) by Eq. (4.15). We conclude that

#### Proposition 4.4

The ABHY scattering form $\Omega _a$ is given by Eq. (4.25).

More generally, given any CHY half-integrand $\mathcal {I}$, we can define an ABHY scattering form

4.27 $$\begin{aligned} \Omega _{\mathcal {I}} := \int _{p^{-1}(s_{ij})} q^* \left[ \bar{\delta }^{n-3}(\underline{\tau }) \wedge \omega ^{n-3} \,\mathcal {I}\right] . \end{aligned}$$

This form admits an expansion

4.28 $$\begin{aligned} \Omega _{\mathcal {I}} = \sum _{a} w_{\Gamma _{1a}} \int _{p^{-1}(s_{ij})} q^* \left[ \bar{\delta }^{n-3}(\underline{\tau }) PT_{1a} \mathcal {I}\right] . \end{aligned}$$

The coefficients of the forms $w_{\Gamma _{1a}}$ in this sum are the BCJ numerators, $N_{1a}$, associated with the integrand $\mathcal {I}$. This formula is in line with earlier work on extracting BCJ numerators from CHY half integrands, especially [37]. The CHY integral as given is not changed by adding to $\mathcal {I}$ a term that vanishes on the support of the scattering equations. Indeed, one can regard $\mathcal {I}$ as a representative of a twisted cohomology class, as explained in [38].

All scattering forms, $\Omega _{\mathcal {I}}$, obtained in this way are projective. Let $\Upsilon$ be the Euler vector field on $\mathcal {K}_n$,

$$\begin{aligned} \Upsilon = \sum _{i<j}s_{ij}\frac{\partial ~}{\partial s_{ij}}. \end{aligned}$$

We can lift $\Upsilon$ trivially to $\mathcal {Y}_n$ using the product structure. On objects pulled back from $\mathbb {T}$, it then acts by

4.29 $$\begin{aligned} \Upsilon = \sum _i \Upsilon (\tau _i)\frac{\partial ~}{\partial \tau _i}=\sum _i\tau _i\frac{\partial ~}{\partial \tau _i}. \end{aligned}$$

Contracting $\Upsilon$ into $\Omega (a)$, we findon account of the delta functions in the integrand.

#### Lemma 4.5

The scattering form $\Omega _{\mathcal {I}}$ is a projective form on $\mathbb {P}\mathcal {K}_n$.

Finally, amplitudes are obtained by restricting the scattering forms $\Omega _{\mathcal {I}}$ to ABHY planes, as in [6]. In fact, the restriction of $\Omega _{\mathcal {I}}$ to $P_a$ evaluates to give the CHY formula for the integrands $PT_a$ and $\mathcal {I}$, as follows from the definition of $P_a$ (Eq. (4.19) and subsequent lines). Moreover, for a general cohomology class

$$\begin{aligned} N := \sum _a N_{1a}PT_{1a} \in H^{n-3}, \end{aligned}$$

we have an associated plane, $P_N := \sum _a N_{1a} P_{1a}$. Pairing this with the general scattering form $\Omega _{\mathcal {I}}$ gives the CHY amplitude for integrands *N* and $\mathcal {I}$.

## Discussion

We have seen that Lie polynomials underpin the colour-kinematics and double copy framework of BCJ. We have reviewed the classical fact that the top homology of $\mathcal {M}_{0,n}-D$ is isomorphic to the Lie polynomials, $Lie(n-1)$, and shown that there is a natural correspondence between $T^*_D\mathcal {M}_{0,n}$ and $\mathcal {K}_n$ under which the CHY integral formulae can be understood as a Penrose transform. This can be extended to a transform between the holomorphic Liouville form and the differential forms $w_\Gamma$ introduced by [6], and also between CHY half-integrands and the scattering forms introduced by [6].

One underlying question in the subject is whether there is a kinematic algebra underpinning the kinematic numerators $N_\Gamma ^{k,\epsilon }$. Although we have seen that the colour factors of Lie algebras can provide such numerators, we have also seen many examples of numerators satisfying the Jacobi identity that do not arise as colour factors for a Lie algebra: for example, $(\Gamma ,a)$ and $w_\Gamma$ and so on. In other words, the existence of a homomorphism from $Lie(n-1)$ to some vector space does not of itself determine a Lie algebra.

The basic results in this paper can be taken further to yield natural recursions in field theory, which lead to both Lie polynomial and ABHY-form-based proofs of the known properties of the field theory momentum kernel and of kinematic numerators. The momentum kernel can be studied also in $T^*\mathcal {M}_{0,n}$, where it arises in the CHY treatment of KLT orthogonality [39]. It also seems likely that the framework will naturally extend to loop integrands in the context of nodal spheres following the logic of [40–44].

The correspondence we have described is suggestive of the naive explicit formula

5.1 $$\begin{aligned} N^{\mathcal {I}_l}_\Gamma =\int _{C_\Gamma }\mathcal {I}_l, \end{aligned}$$

for numerators in terms of CHY half-integrands. However, such formulae fail for the CHY Pfaffian that one would expect to give the basic Yang–Mills kinematic numerators; the formula is compromised by the interdependence between in particular reduced Pfaffians and scattering equations. This equation is shown to be invalid as written but a slightly different formulation in a similar spirit is shown to work when $\mathcal {I}_l$ represents a cohomology class in the context of twisted cohomology by Mizera [45].

We remark that the twisted cycle formulation of string integrals in [46] naturally arises in the context of the holomorphic geometric quantization of $T^*_D\mathcal {M}_{0,n}$. To carry out geometric quantization, one introduces the line bundle $\mathcal {L}\rightarrow T^*_D\mathcal {M}_{0,n}$ with connection $\nabla =d+ \alpha '\tau$ where $\tau =\sum _i \tau _i d\sigma _i$ is the canonical 1-form (symplectic potential) and $\alpha '$ plays the role of Planck’s constant. Polarized wave functions should be independent of $\tau _i$. On pull back to the correspondence space, $\mathcal {Y}_n$, the connection $\nabla$ becomes the standard twisted exterior derivative associated with the Koba–Nielsen factor. Such a quantization of $T^*\mathcal {M}_{0,n}$ perhaps most naturally arises from ambitwistor-string path-integral [47], where the Pfaffian half-integrand for kinematic numerators arises from the RNS spin field path-integral.

There are many further connections to be followed up; we briefly mention the delta algebras of [48] and the formulations of colour-kinematics duality in [49, 50].

## Footnotes

1 The shuffle product of *a* and *b*, , is the sum over ordered permutations of the letters of *a* and *b* that preserve the orderings of the letters in *a* and *b*.

2 One way to avoid the Gram conditions is to suppose that the $k_i^\mu$ are *n* vectors in $(n-1,1)$ dimensional Minkowski space. However, the momentum conservation relation, $\sum k_i^\mu = 0$, together with the null conditions, $k_i^2 = 0$, imply a further determinantal condition on the $s_{ij}$ variables, even in $d\ge n$ dimensions.

3 This is eq 3.8 of [15] together with the use of the *U*(1)-decoupling identity in the bracketed term.

4 Arnol’d [21] computes the Poincaré polynomial of the cohomology of the configuration space $M_{n-1}$ of $n-1$ points in $\mathbb {C}$. This can be obtained inductively via the fibration $M_{k}\rightarrow M_{k-1}$ with fibre $\mathbb {C}-\{\sigma _1,\ldots ,\sigma _{k-1}\}$ giving factors of $(1+(k-1)t)$. To get $\mathcal {M}_{0,n}^\#$, one needs to take the quotient of $M_{n-1}$ by $\mathbb {C}^*\ltimes \mathbb {C}$, the stabilizer in $PSL_2$ of the point at infinity. Arnol’d’s formula for the Poincaré polynomial of $M_{n-1}$ must therefore be divided by $1+t$ to obtain the formula here.

5 We thank Carlos Mafra for this reference and also refer the reader to sections 4.1. and 4.2 of [22] for further discussion.

6 There is a free action of the symmetric group $S_n$ on $H_{n-3}(\mathcal {M}_{0,n}-D)$. As a module for $S_n$, $H_{n-3}(\mathcal {M}_{0,n}-D)$ is not isomorphic to $Lie(n-1)$, but $Lie(n-1)$ twisted by the sign representation of $S_n$.

7 The homology of configuration spaces was first computed by [28], but the cycles are described by Cohen [27]. Cohen was studying the homology of configuration spaces. To apply his results here, one uses the isomorphism $\mathcal {M}_{0,n}-D \simeq \text {Conf}_{n-3}(\mathbb {R}^2-\{0,1\})$.

8 This is a consequence of Theorem 1.2 in [27], as reviewed in [30].

9 The maximum dimension tori appearing in Lemma 3.3 can be constructed by taking the wedge products of lower dimensional tori. This leads to a natural product in the full homology $H_*(\mathcal {M}_{0,n}-D)$, under which $H_*$ can be identified with a graded Lie algebra, with graded Jacobi relations holding in all dimensions. [27] The (ungraded) Jacobi relation in Lemma 3.3 is a special case [27, 30].

10 For completeness, the CHY Pfaffian is the reduced Pfaffian of the matrix *M*

$$\begin{aligned} M=\begin{pmatrix}A&{}C\\ -C^t&{}B\end{pmatrix} ,\quad A_{ij}=\frac{k_i\cdot k_j}{\sigma _i-\sigma _j}, \quad B_{ij}=\frac{\epsilon _{i}\cdot \epsilon _{j}}{\sigma _i-\sigma _j} , \quad C_{ij}=\frac{k_i\cdot \epsilon _{j}}{\sigma _i-\sigma _j}, \quad \text{ for } i\ne j \end{aligned}$$

constructed from polarization vectors $\epsilon _{i}$ with $k_i\cdot \epsilon _{i}=0 \ldots$, and $A_{ii}=B_{ii}=0$, $C_{ii}=\epsilon _{i}\cdot \sum k_i/\sigma _{ij}$. This matrix is degenerate, but removing two rows and columns, say *ij*, taking the Pfaffian and dividing by $\sigma _{ij}$ yields a well-defined half-integrand.
